# Evaluating a 6.0-mm candidate warning level for intraoperative L5 nerve root retraction distance during posterior lumbar interbody fusion: a retrospective cohort study

**DOI:** 10.1186/s12891-026-10356-4

**Published:** 2026-08-14

**Authors:** Min Wang, Jixin Li, Zhiqing Chen, Jie Lan, Shanglong Ning, Qiang Yang, Jun Miao, Bangquan Liao

**Affiliations:** 1 Department of Orthopedics (Spine Division), Beichen Hospital, Nankai University, No. 7 Beiyi Road, Beichen District, Tianjin, China; 2 Clinical School, College of Orthopedics, Tianjin Medical University, No. 22 Qixiangtai Road, Heping District, Tianjin, China; 3 Clinical College of Orthopedics, Tianjin Medical University, 406 Jiefang South Road, Hexi District, Tianjin, China; 4 Department of Spine Surgery, Tianjin Hospital, Tianjin University, 406 Jiefang South Road, Hexi District, Tianjin, China; 5 School of Physical Science and Technology, Tiangong University, Tianjin, China

**Keywords:** Posterior lumbar interbody fusion, Radiculitis, Nerve root retraction, Safety threshold, Surrogate marker, Dose-response relationship.

## Abstract

**Background:**

Postoperative radiculitis secondary to excessive L5 nerve root retraction is a debilitating complication of posterior lumbar interbody fusion (PLIF). Despite its clinical prevalence, surgeons lack a standardized, visual safety threshold, often relying on intraoperative experience and tactile feedback. This study utilized maximum physical retraction distance as a practical but incomplete proxy for mechanical nerve stress to explore a potential risk threshold and evaluate its mid-term clinical impact.

**Methods:**

In this retrospective cohort study, we analyzed 80 patients who underwent PLIF. All procedures were performed using a standardized technique to ensure consistency. The maximum intraoperative transverse retraction distance of the L5 nerve root was recorded. The primary endpoint was the development of postoperative radiculitis. The optimal diagnostic cutoff was determined via receiver operating characteristic (ROC) analysis. A Cochran-Armitage test assessed the dose-response relationship, and 6-month visual analog scale (VAS), Oswestry Disability Index (ODI), and reintervention rates were evaluated.

**Results:**

Postoperative radiculitis developed in 18 of 80 patients (22.5%). Multivariable regression confirmed maximum retraction distance as a robust independent predictor. ROC analysis established a 6.0 mm diagnostic cutoff (AUC = 0.678, *P* = 0.010; Negative Predictive Value = 87.2%). Retraction > 6.0 mm almost tripled the radiculitis rate compared to ≤ 6.0 mm (36.4% vs. 12.8%; RR = 2.85, 95% CI: 1.19–6.82, *P* = 0.013), yielding a massive population attributable risk (PAR) of 43.3%. Furthermore, the injury risk escalated linearly with wider displacement (*P* = 0.011). By 6 months, affected patients exhibited significantly worse VAS and ODI scores (*P* < 0.01, Cohen’s d > 0.8), necessitating substantially more additional interventions (RR = 2.76) and reoperations (RR = 3.44).

**Conclusions:**

Transverse L5 nerve root retraction exceeding 6.0 mm is associated with an elevated risk of postoperative radiculitis, significantly impairing mid-term clinical outcomes. In standard surgical settings lacking real-time force-sensing equipment, restricting nerve displacement to ≤ 6.0 mm provides surgeons with a practical candidate warning level.

**Supplementary Information:**

The online version contains supplementary material available at https://doi.org/10.1186/s12891-026-10356-4.

## Background

Posterior lumbar interbody fusion (PLIF) reliably addresses lumbar degenerative pathology, yet iatrogenic radiculitis frequently complicates postoperative recovery. Reported incidence rates vary widely across studies, spanning from 0.9% up to 44% [1–3]. Beyond simply prolonging hospital admissions, this neurological decline can manifest as permanent sensorimotor deficits—effectively negating the intended benefits of the index surgery [4]. Excessive intraoperative nerve root retraction is well-documented as the main driver behind this preventable morbidity [5–7]. Consequently, spine surgeons face a constant technical trade-off: achieving adequate interbody exposure without over-stretching the neural elements. Quantifying an objective safety threshold for retraction is therefore essential to minimize surgical risk.

Anatomical studies reveal that the L5 nerve root exits the dural sac at a steeper angle and passes through a relatively narrow bony foraminal canal. The dorsal root ganglion (DRG) is frequently located within the intervertebral foramen, restricting its mobility [8, 9]. These anatomical traits noticeably lower its tolerance to mechanical retraction and displacement. Basic biomechanical and animal studies confirm that maintaining the structural and functional integrity of neural tissues depends on clear biomechanical thresholds. When retraction displacement or tension exceeds specific limits [10, 11], or when the duration is too long [5, 12], it rapidly blocks blood perfusion within the nerve root and halts axonal transport. This ultimately causes demyelination and irreversible microstructural damage [13, 14], cascading into a series of neuroinflammatory responses [10, 15, 16]. 

Intraoperative neuromonitoring (IONM) has seen widespread use in spine surgery in recent years. However, high equipment costs and the need for specialized neurophysiologists limit its routine use in primary healthcare facilities [17–19]. Furthermore, IONM shows a noticeable time lag in detecting mechanical retraction of lumbar nerve roots [20, 21]. Somatosensory evoked potentials (SSEPs) have a sensitivity of only 13.6% for motor deficits, while spontaneous electromyography (s-EMG) sits at 48.3%, indicating a risk of false negatives [18, 22]. At the same time, electrophysiological signals are easily skewed by factors like anesthetics, body temperature, and intraoperative hemodynamic shifts. This complicates monitoring and compromises timely warning judgments [23]. Consequently, finding an intuitive, universally applicable monitoring method that does not rely on electrophysiological equipment carries major practical significance for standardizing grassroots surgical procedures.

In recent explorations, several studies have utilized custom force-sensing devices (such as piezoresistive retractors, MEMS modules, and specialized dynamometers) to perceive intraoperative nerve root stress. These have confirmed an intrinsic link between mechanical stress and postoperative nerve injury [24–26]. Yet, challenges like bulky device size, susceptibility to interference, cable dependency, and difficulties in clinical adaptation still hinder their clinical translation. To address this, our team previously applied Fiber Bragg Grating (FBG) sensing technology to conduct in vitro biomechanical validations of nerve root retraction force and distance. The results showed that, simulating the transverse retraction of the nerve root during PLIF, there is a clear linear correlation between the transverse retraction distance and the deformation force at the tip of the retractor (see Supplementary Figs. 1 and 2). This demonstrates that, without real-time in vivo force-sensing equipment, the directly measurable intraoperative retraction distance can serve as a reliable clinical surrogate for the micro-mechanical stress state of the nerve root.

Currently, in routine PLIF procedures, the magnitude of nerve root retraction still relies on the surgeon’s subjective experience and tactile feedback. There remains a lack of large-sample, evidence-based quantitative standards for a safe retraction distance. This study aimed to use a custom nerve root measurement device to objectively record the maximum transverse displacement of the L5 nerve root during surgery. By quantitatively analyzing its correlation with postoperative radiculitis, we sought to explore a safe retraction boundary for surgical operations. More importantly, we attempted to verify whether this accessible and highly applicable physical measurement tool could provide a rapidly replicable data collection method for future multi-center, large-sample studies on preventing nerve injury.

## Materials and methods

### Study design and population

Approved by our institutional Medical Ethics Committee and adhering strictly to STROBE guidelines, this retrospective cohort study reviewed 80 patients (aged 40–75 years) treated between September 2023 and December 2024. All subjects underwent single-level L4/5 posterior lumbar interbody fusion (PLIF) for lumbar spinal stenosis, with or without Meyerding [27] grade I-II spondylolisthesis.

Cohort assembly relied on strict data availability. Specifically, every included patient required precise L5 nerve root retraction distances documented in their operative notes, combined with an uninterrupted 6-month clinical observation period (with clinical visits at 1, 3, and 6 months). Zero patients dropped out during this follow-up phase. Exclusion criteria restricted patients with prior lumbar operations, severe baseline L5 nerve impairment (Medical Research Council [MRC] [28] muscle strength ≤ 3), or missing data points.

### Surgical technique and retraction measurement

Two senior spine surgeons performed all procedures using standard posterior PLIF techniques with unilateral cage placement. During the active discectomy and cage insertion phases, we recorded the maximum transverse retraction displacement of the L5 nerve root utilizing a custom-built, micrometer-based measurement device capable of an accuracy of ± 0.01 mm. Prior to routine sterilization, the instrument’s accuracy was strictly calibrated against standard gauge blocks.

**Fig. 1:**
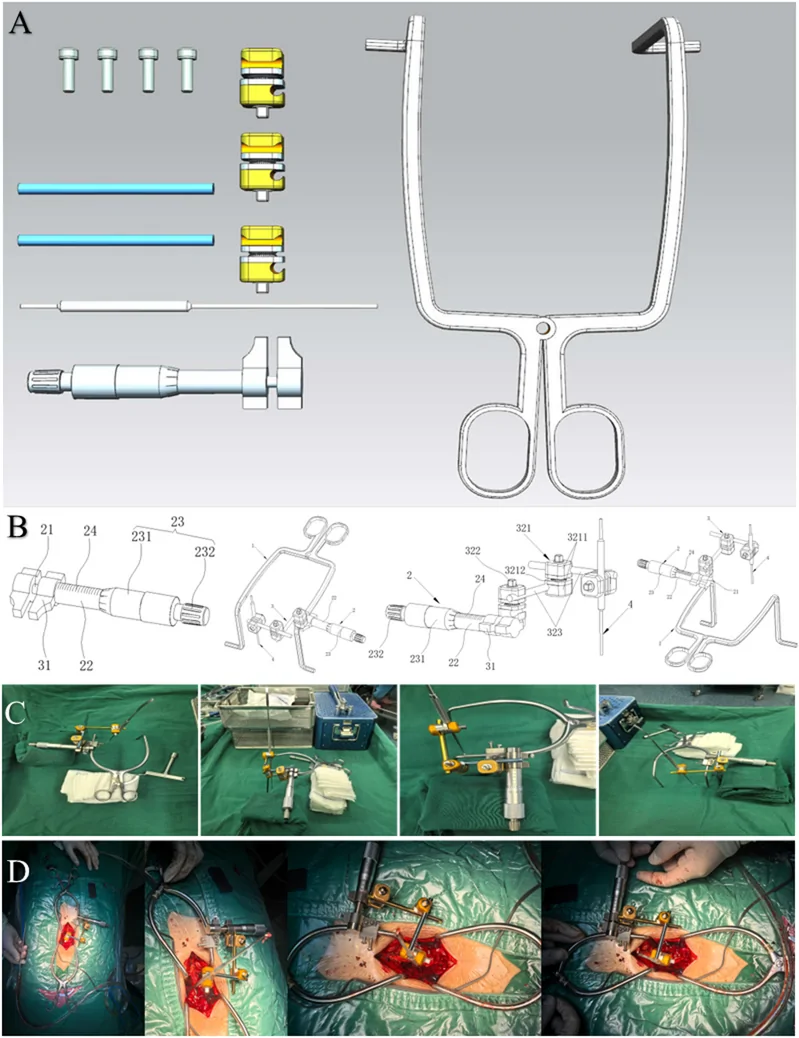
Custom micrometer-based device for measuring L5 nerve root retraction. **A** Physical photograph of the measurement instrument. **B** Schematic illustration detailing the device design. **C** Sterile presentation of the instrument on the surgical table prior to application. **D** Intraoperative view demonstrating the retractor tip securely docked against the L5 nerve root shoulder to record maximum transverse displacement

In practice, the custom measurement device (physical view, Fig. 1A; schematic design, Fig. 1B; sterile intraoperative appearance on the surgical table, Fig. 1C) was introduced to the field after the nerve root was sufficiently skeletonized. The surgeon established a zero baseline by docking the retractor tip directly against the shoulder of the L5 nerve root. An intermittent retraction technique was routinely applied throughout the procedures. We precisely recorded the maximum transverse retraction distance and the cumulative retraction time (intraoperative measurement, Fig. 1D). All operative maneuvers and the magnitude of retraction relied entirely on the surgeons’ standard clinical experience.

### Definition of outcome measures

A formal diagnosis of L5 radiculitis was established if patients developed—or experienced a distinct worsening of—at least one of the following neurological deficits postoperatively: (1) escalated radicular pain strictly mapping to the L5 dermatome; (2) newly identified motor weakness in L5-innervated muscle groups (e.g., tibialis anterior or extensor hallucis longus); (3) progressive sensory blunting, including profound numbness or hypesthesia; and (4) absence of structural etiologies (such as cage migration, misplaced screws, or epidural hematoma) verified on postoperative imaging. In this study, “radiculitis” was utilized as a broad clinical umbrella term encompassing both transient perioperative irritation (neuropraxia) and persistent radicular symptoms, as both reflect meaningful mechanical stress and edema directly induced by intraoperative retraction.

Secondary endpoints evaluated at the 6-month mark comprised visual analog scale (VAS) scores, the Oswestry Disability Index (ODI), and subjective patient satisfaction. To minimize ascertainment bias, all postoperative clinical outcomes were retrospectively extracted and adjudicated by an independent investigator blinded to the intraoperative retraction measurements.

### Statistical analysis

Data workup relied on R software (version 4.5.1), with two-sided *P* values < 0.05 defining statistical significance. The analytical framework proceeded in three stages:

1. Association and Risk Factors: Model selection utilized a bidirectional stepwise algorithm guided by the Akaike Information Criterion (AIC). After screening for multicollinearity via Variance Inflation Factors (VIF) and checking residuals, we ran multivariate logistic regressions. Given the sample size, Firth’s penalized likelihood estimation was incorporated into the modeling to adjust for potential bias. The Hosmer-Lemeshow test and C-index provided metrics for goodness-of-fit.
2. Diagnostic Thresholds: The discriminative ability of the retraction distance was evaluated via receiver operating characteristic (ROC) models. By maximizing the Youden Index across the area under the curve (AUC), an optimal diagnostic cut-off emerged. This specific threshold yielded the corresponding sensitivity, specificity, alongside both positive and negative predictive values (PPV/NPV).
3. Validation and Robustness: For stratified analysis, the continuous retraction data were partitioned into four predefined brackets (≤ 4.0, 4.1–6.0, 6.1–8.0, and > 8.0 mm) to assess dose-response trends via the Cochran-Armitage test. Internal model validation utilized a 1000-iteration Bootstrap resampling technique to correct for overfitting. We subsequently challenged the model’s stability through sensitivity analyses across adjacent parameters (such as comparing 6.0 mm against 7.0 mm).

## Results

### Study population and surgical characteristics

Eighty adults were included in the study group (18 with and 62 without postoperative radiculitis). No significant differences were observed between the two groups regarding age, gender, BMI, comorbidities, or preoperative clinical scores (all *P* > 0.05, Table 1). The measurements of cumulative retraction time demonstrated high consistency between the groups (*P* = 0.412). However, the maximum L5 retraction distance in the radiculitis group was significantly greater than that in the non-radiculitis group (6.64 ± 1.48 mm vs. 5.61 ± 1.55 mm; *P* = 0.015).

**Table 1:** Baseline Characteristics and Surgical Parameters of the Study Population

Characteristics	No Radiculitis (*n* = 62)	Radiculitis (*n* = 18)	*P* value
Demographics			
Age (years)	56.52 ± 6.40	59.44 ± 7.63	0.106
Gender (Male/Female)	28 (45.2%) / 34 (54.8%)	11 (61.1%) / 7 (38.9%)	0.289†
BMI (kg/m²)	24.39 ± 1.81	24.53 ± 1.41	0.773
Diabetes mellitus (Yes/No)	8 (12.9%) / 54 (87.1%)	2 (11.1%) / 16 (88.9%)	1.000†
Clinical Assessment			
Preoperative VAS back pain	6.85 ± 0.77	7.21 ± 0.69	0.081
Preoperative VAS leg pain	7.15 ± 0.94	7.45 ± 0.85	0.233
Preoperative ODI (%)	41.90 ± 5.25	42.53 ± 4.35	0.644
Tibialis anterior strength (Grade IV/V) ‡	19 (30.6%) / 43 (69.4%)	3 (16.7%) / 15 (83.3%)	0.370†
L5 dermatome sensation§ (Abnormal/Normal)	15 (24.2%) / 47 (75.8%)	4 (22.2%) / 14 (77.8%)	1.000†
Surgical Parameters			
Operative time (min)	138.03 ± 15.23	140.89 ± 22.25	0.532
Estimated blood loss (mL)	165.42 ± 24.55	176.22 ± 26.67	0.111
Retraction time (min)	10.32 ± 1.84	10.74 ± 2.15	0.412
L5 retraction distance (mm)	5.61 ± 1.55	6.64 ± 1.48	0.015*

Values are mean ± SD or n (%)

† Continuous variables compared via independent t-test (Welch’s correction applied); categorical variables via Fisher’s exact test. ‡ Evaluated by the Medical Research Council (MRC) grading system. § Defined as any sensory deficit or paresthesia within the L5 dermatome; * *P* < 0.05

### Logistic regression analysis of risk factors for postoperative radiculitis

**Fig. 2:**
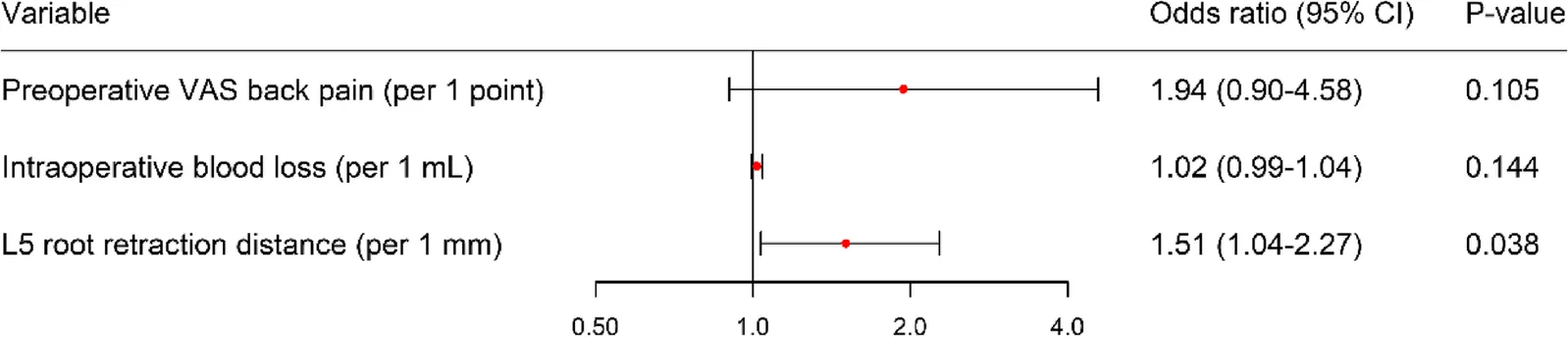
Forest plot detailing the multivariable logistic regression for postoperative L5 radiculitis. We optimized this model via bidirectional stepwise regression guided by the Akaike Information Criterion (AIC). Notably, maximum L5 retraction distance emerged as a significant independent risk factor (*P* = 0.038). Abbreviations: OR, odds ratio; CI, confidence interval; VAS, Visual Analog Scale

**Fig. 3:**
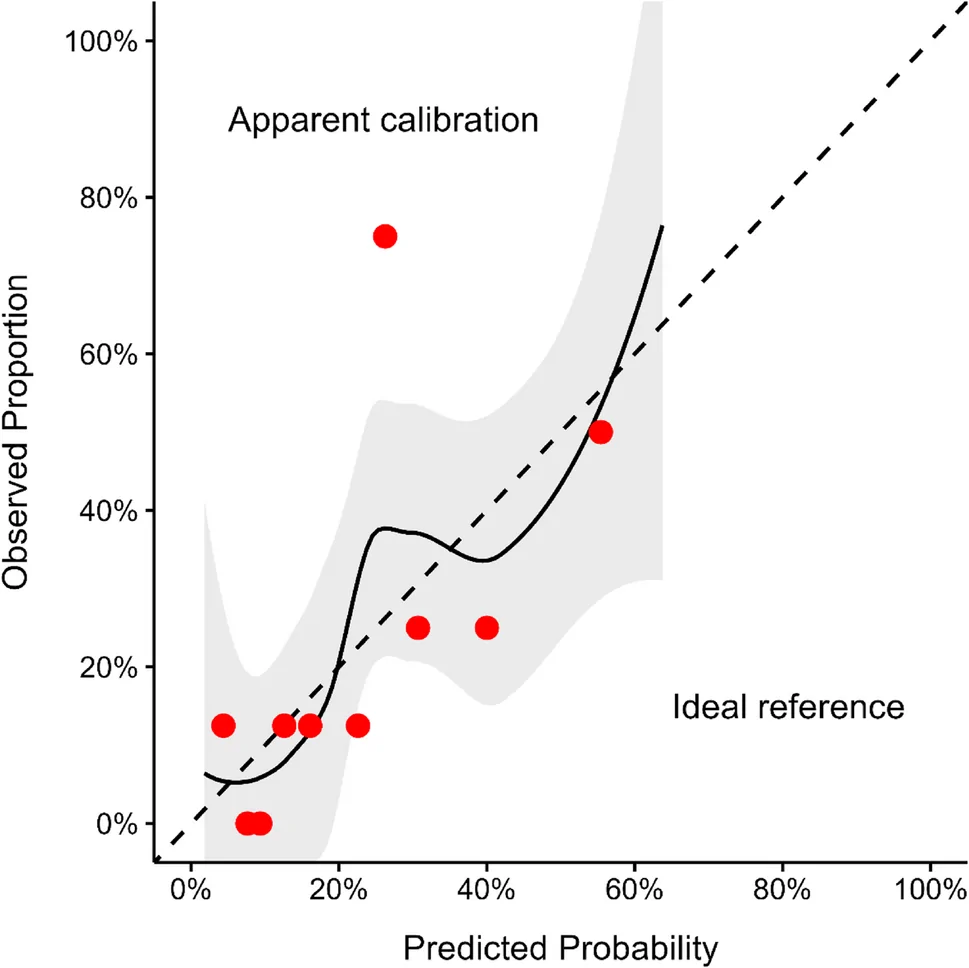
Calibration plot assessing the multivariable predictive model. The dashed diagonal line illustrates a perfectly calibrated ideal reference, whereas the solid line maps the actual diagnostic performance of our current model. Each red dot groups a decile of predicted probabilities. The model demonstrated reliable goodness-of-fit (Hosmer-Lemeshow test, *P* = 0.080)

Univariate logistic regression identified the maximum L5 retraction distance as significantly associated with postoperative radiculitis (OR = 1.55; 95% CI, 1.09–2.30; *P* = 0.020). To account for potential confounders, clinically relevant variables were incorporated into a multivariable model optimized via the Akaike Information Criterion (AIC). After adjusting for preoperative VAS back pain and intraoperative blood loss, the maximum L5 retraction distance remained an independent risk factor for radiculitis (adjusted OR = 1.51; 95% CI, 1.04–2.27; *P* = 0.038; Table 2; Figs. 2 and 3). Multicollinearity diagnostics confirmed the independence of included variables (VIF < 1.05).

The model demonstrated favorable discriminative power (C-index = 0.739) and adequate calibration (Hosmer-Lemeshow test, *P* = 0.080; Fig. 3). Given the limited cohort size, Firth’s penalized likelihood method was employed to control for potential overfitting bias. The corrected results remained robust, confirming the significant impact of retraction distance (OR = 1.46; 95% CI, 1.02–2.16; *P* = 0.037; Supplementary Table 1).

**Table 2:** Univariate and multivariable logistic regression analysis of risk factors for postoperative radiculitis

Variables	Univariate Analysis		Multivariable Analysis†	
	OR (95% CI)	*P* value	OR (95% CI)	*P* value
Core Predictive Parameter				
L5 root retraction distance (mm)	1.55 (1.09–2.30)	0.020*	1.51 (1.04–2.27)	0.038*
Demographics and Clinical Characteristics				
Age (year)	1.07 (0.99–1.16)	0.109	—	—
Gender (Male vs. Female)	1.89 (0.68–5.25)	0.224	—	—
BMI (kg/m²)	1.05 (0.77–1.43)	0.770	—	—
Diabetes mellitus (Yes vs. No)	0.85 (0.17–4.20)	0.843	—	—
Preoperative VAS back pain	1.95 (0.94–4.40)	0.085	1.94 (0.90–4.58)	0.105
Preoperative VAS leg pain	1.43 (0.80–2.64)	0.232	—	—
Preoperative ODI (%)	1.02 (0.94–1.11)	0.610	—	—
Tibialis anterior strength (Grade IV/V)	2.21 (0.57–8.54)	0.251	—	—
L5 dermatome sensation (Normal vs. Abnormal)	1.12 (0.32–3.91)	0.863	—	—
Surgical Parameters				
Operative time (min)	1.01 (0.98–1.04)	0.527	—	—
Intraoperative blood loss (mL)	1.02 (1.00–1.04)	0.114	1.02 (0.99–1.04)	0.144
Retraction time (min)	1.12 (0.85–1.48)	0.408	—	—

The multivariable logistic regression model was optimized using bidirectional stepwise selection based on the Akaike Information Criterion (AIC). Variables marked with “—” did not enter the final optimal model. Variance Inflation Factor (VIF) for all included variables was < 1.05, indicating no multicollinearity. Model performance metrics: C-index = 0.739 (95% CI: 0.613–0.866); Hosmer-Lemeshow goodness-of-fit test *P* = 0.080; Nagelkerke *R*² = 0.263;Statistically significant difference (*P* < 0.05)

### Diagnostic efficacy and cutoff analysis for L5 retraction distance

**Fig. 4:**
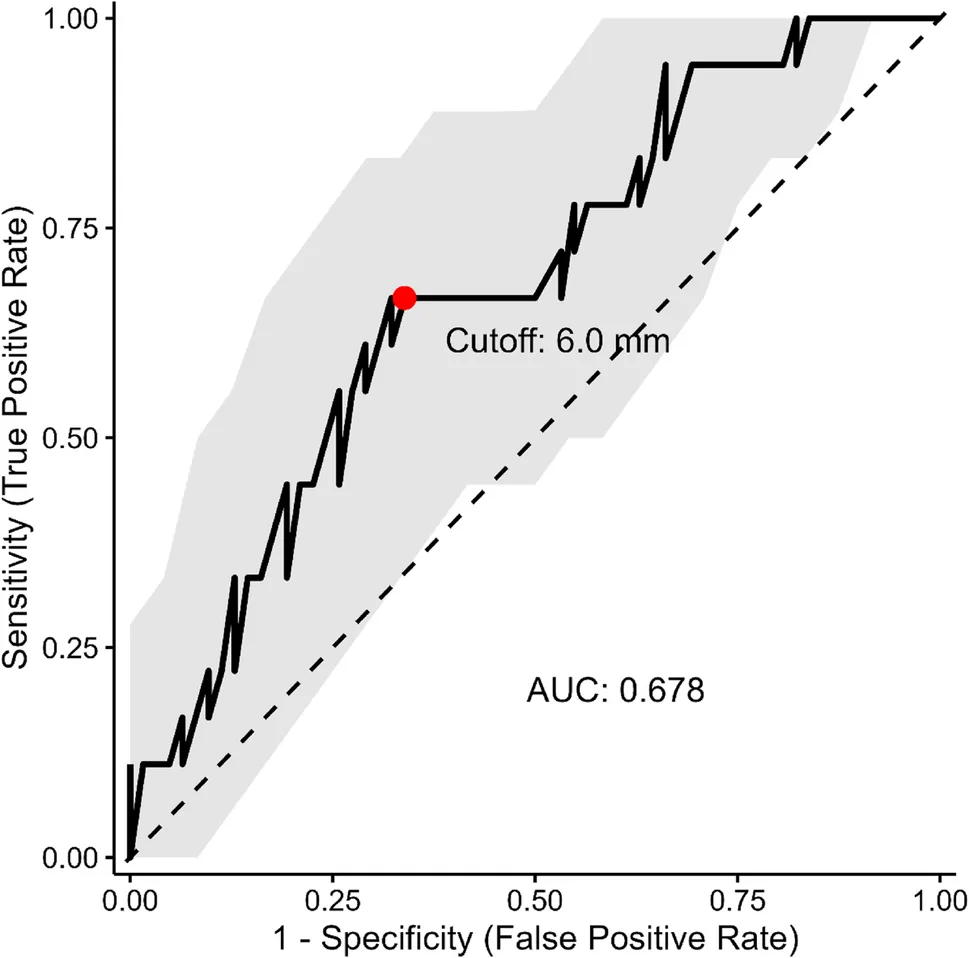
Receiver operating characteristic (ROC) analysis evaluating L5 retraction distance. The solid black curve charts the diagnostic performance, yielding an area under the curve (AUC) of 0.678. Gray shading outlines the 95% confidence interval. We pinpointed the 6.0 mm optimal cutoff (marked by the red dot) by maximizing the Youden index

The discriminative ability for predicting postoperative radiculitis was significant for the maximum L5 retraction distance, with an area under the curve (AUC) of 0.678 (95% CI: 0.541–0.816, *P* = 0.010). This predictive performance remained robust after internal validation using 1,000 bootstrap replicates (bias-corrected AUC = 0.679). According to the Youden index, the optimal diagnostic cutoff for the L5 retraction distance was 6.0 mm, yielding a sensitivity of 66.7%, a specificity of 66.1%, and a negative predictive value (NPV) of 87.2%. In the sensitivity analysis, reducing the cutoff threshold to 5.0 mm increased the sensitivity to 88.9% and the NPV to 91.3% (Table 3; Fig. 4).

**Table 3:** Diagnostic performance of L5 root retraction distance for predicting postoperative radiculitis

Metric	Value (95% CI)
Overall Performance	
Area Under the Curve (AUC)	0.678 (0.541–0.816)*
Bootstrap-corrected AUC†	0.679 (0.527–0.807)
Optimal Cutoff: 6.0 mm‡	
Sensitivity (%)	66.7 (41.0–86.7)
Specificity (%)	66.1 (53.0–77.7)
Positive Predictive Value (%)	36.4 (20.4–54.9)
Negative Predictive Value (%)	87.2 (74.3–95.2)
Positive Likelihood Ratio	2.0 (1.2–3.2)
Negative Likelihood Ratio	0.5 (0.3–1.0)
Youden Index	0.328
High-Sensitivity Cutoff: 5.0 mm	
Sensitivity (%)	88.9 (65.3–98.6)
Specificity (%)	33.9 (22.3–47.0)
Negative Predictive Value (%)	91.3 (72.0–98.9)

Compared with null hypothesis (0.5) via DeLong’s test (*P* = 0.010)

†Bias-corrected estimates and internal validation derived from 1,000 bootstrap replicates

‡Optimal cutoff determined by maximizing the Youden Index

### Risk stratification and dose-response relationship analysis

Risk stratification analysis revealed that the incidence of postoperative L5 radiculitis increased progressively with greater retraction distances. Specifically, no radiculitis occurred when the retraction distance was ≤ 4.0 mm (0% incidence), whereas the incidence rose to 37.0% as the distance increased to 6.1–8.0 mm (Table 4).The Cochran-Armitage trend test confirmed a statistically significant dose-response relationship between retraction magnitude and radiculitis risk (Z = 2.55, *P* = 0.011).

**Fig. 5:**
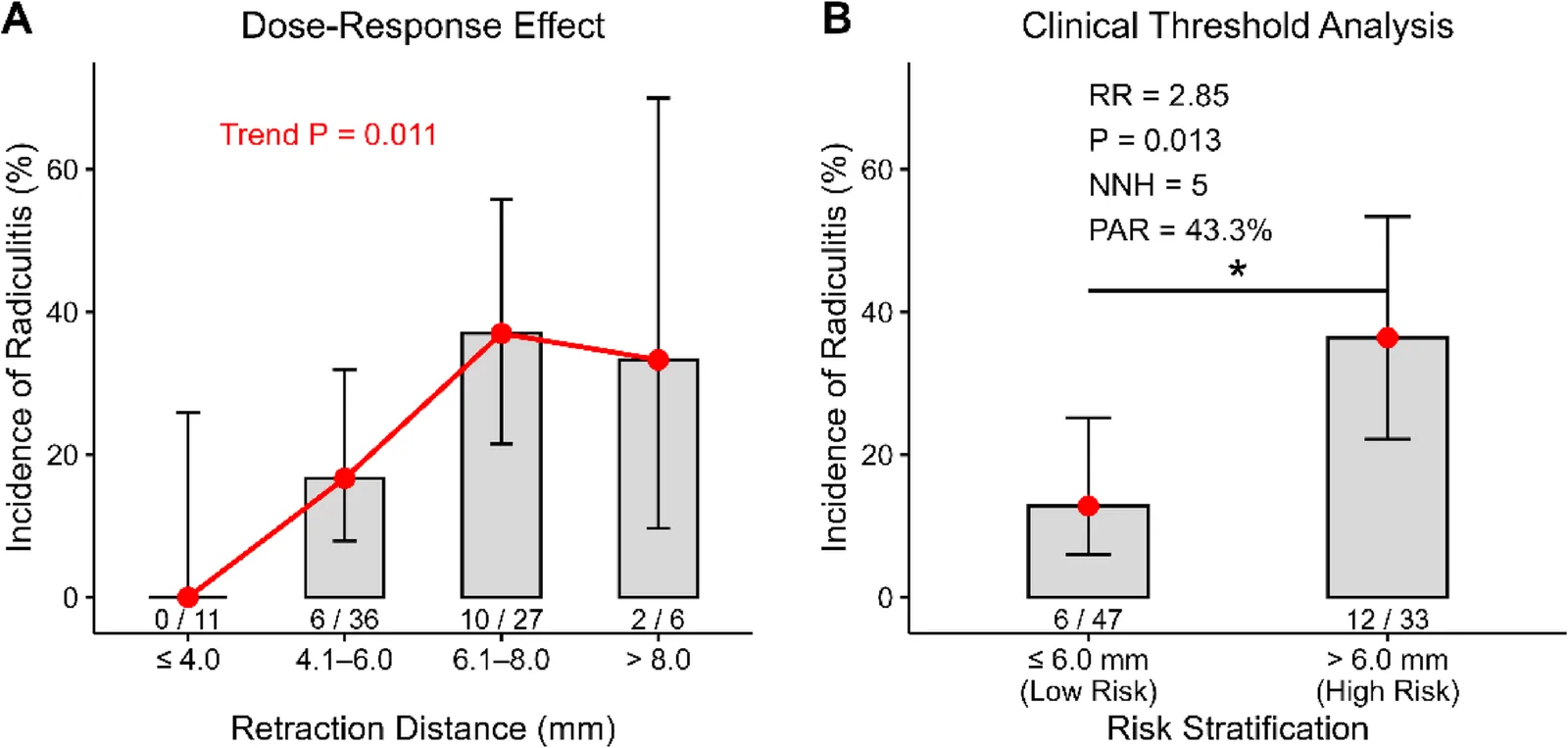
Dose-response relationship and clinical threshold analysis for retraction distance. **A** Postoperative radiculitis incidence stratified across four ordinal displacement brackets. The overlaid red line highlights a statistically significant linear trend (*P* = 0.011). **B** Risk comparison dichotomized by the 6.0 mm safety boundary. Patients exceeding this threshold faced a sharply elevated risk (*P = 0.013). Error bars display 95% confidence intervals. Abbreviations: RR, relative risk; NNH, number needed to harm; PAR, population attributable risk

Further clinical threshold analysis established 6.0 mm as the critical cutoff point. Patients with a retraction distance > 6.0 mm exhibited a radiculitis incidence of 36.4%, which was significantly higher than the 12.8% observed in the ≤ 6.0 mm group (*P* = 0.013). The corresponding relative risk (RR) was 2.85 (95% CI: 1.19–6.82), with a number needed to harm (NNH) of 5 and a population attributable risk (PAR) of 43.3% (Fig. 5).

**Table 4:** Dose-response relationship and clinical threshold analysis of L5 root retraction distance and postoperative radiculitis

Retraction Distance	Cases / Total	Incidence Rate, % (95% CI)	Relative Risk (95% CI)	*P* Value
Dose-response analysis				
≤ 4.0 mm	0 / 11	0.0 (0.0–25.9)	1.00 (Reference)	—
4.1–6.0 mm	6 / 36	16.7 (7.9–31.9)	2.17 (0.29–16.33)	—
6.1–8.0 mm	10 / 27	37.0 (21.5–55.8)	4.81 (0.69–33.72)	—
> 8.0 mm	2 / 6	33.3 (9.7–70.0)	4.33 (0.48–38.99)	—
*Trend test*			*Z* = 2.55	0.011
Clinical threshold analysis				
≤ 6.0 mm (Low risk)	6 / 47	12.8 (6.0–25.2)	1.00 (Reference)	—
> 6.0 mm (High risk)	12 / 33	36.4 (22.2–53.4)	2.85 (1.19–6.82)	0.013*

The 95% CIs for incidence rates were calculated using the Wilson score method

For the dose-response analysis, a continuity correction (adding 1 to cases and 2 to totals) was applied to calculate the relative risk due to zero events in the reference group (≤ 4.0 mm)

*Calculated using the uncorrected Pearson’s Chi-square test. Clinical risk metrics based on the > 6.0 mm threshold: Number Needed to Harm (NNH) = 5; Population Attributable Risk (PAR) = 43.3%

### Clinical outcomes and resource utilization at 6-month follow-up

At the 6-month follow-up, patients in the radiculitis group demonstrated inferior clinical recovery in terms of pain and functional impairment compared to the control group. Regarding VAS scores for back and leg pain, the effect sizes (Cohen’s d) between the two cohorts were 0.930 and 0.938, respectively (both *P* < 0.01). Similarly, the Oswestry Disability Index (ODI) remained significantly higher in the radiculitis group than in the control group (25.71% vs. 17.56%, *P* = 0.008).

In terms of social reintegration and healthcare resource utilization, the radiculitis group exhibited a negative trend; however, these categorical secondary outcomes (e.g., return to work, satisfaction, reoperation) did not reach statistical significance and should be interpreted cautiously as exploratory findings due to limited statistical power. The return-to-work rate was lower in the radiculitis group (66.7% vs. 87.1%, *P* = 0.073), accompanied by a reduction in patient satisfaction (72.2% vs. 85.5%). Furthermore, patients with radiculitis required additional interventions at a rate 2.76 times higher than the control group (22.2% vs. 8.1%). The reoperation rate was also elevated at 16.7% compared to 4.8% in the control group, representing a relative risk (RR) of 3.44 (Table 5).

**Table 5:** Comparison of clinical outcomes at 6-month follow-up between patients with and without postoperative radiculitis

Clinical Outcomes	No Radiculitis (*n* = 62)	Radiculitis (*n* = 18)	Effect Size (MD / RD / RR)*	*P* Value
Continuous variables	Mean ± SD	Mean ± SD	Cohen’s *d*	
VAS Back Pain	2.16 ± 1.41	3.46 ± 1.34	0.930	0.001†
VAS Leg Pain	1.80 ± 1.15	2.93 ± 1.35	0.938	0.004†
ODI (%)	17.56 ± 10.05	25.71 ± 10.64	0.801	0.008†
Categorical variables	n (%)	n (%)	RR (RD, %)	
Return to work	54 (87.1)	12 (66.7)	0.77 (-20.4)	0.073
Patient satisfaction‡	53 (85.5)	13 (72.2)	0.84 (-13.3)	0.288
Extra intervention§	5 (8.1)	4 (22.2)	2.76 (14.2)	0.109
Reoperation	3 (4.8)	3 (16.7)	3.44 (11.8)	0.124

*VAS* Visual Analog Scale, *ODI* Oswestry Disability Index, *RR* Relative Risk, *RD* Risk Difference

†*P* < 0.05, Effect sizes: Cohen’s *d* (continuous), RR (categorical), ‡Satisfied/highly satisfied. §Physical therapy, pain management, or pharmacotherapy

## Discussion

This retrospective cohort study quantitatively reveals the association between L5 nerve root retraction distance during PLIF and the incidence of postoperative radiculitis, preliminarily establishing 6.0 mm as a clinically significant warning threshold. Dose-response analysis demonstrated that retraction exceeding 6.0 mm increases the risk of radiculitis nearly threefold (RR = 2.85). Furthermore, the population attributable risk (PAR) of 43.3% indicates that strictly implementing quantified retraction control intraoperatively could theoretically prevent nearly half of these occurrences. Although retraction-induced injury is a complex, multifactorial biomechanical process, physical displacement is the most direct and easily perceivable metric in the surgeon’s operative field. With continued validation through large-sample, multicenter data, this straightforward and intuitive quantification strategy can be readily popularized and applied, particularly in grassroots hospitals.

Anatomically, as the L5 nerve root exits the dural sac and traverses the lateral recess, it is firmly anchored by the nerve root sleeve and surrounding fibrous tissue, resulting in extremely limited mobility. When the retraction displacement reaches 6.0 mm, it likely approaches the critical tension limit constrained by these anatomical fixation points. The stress generated by this physical displacement likely affects the dorsal root ganglion (DRG) and may potentially induce microcirculatory disturbance. This clinical finding aligns with basic research by Dezawa et al., [29] which demonstrated that mechanical retraction causes acute ischemia of the nerve root. Rat models have similarly shown that when the nerve root is subjected to pressure exceeding 70 g/cm², a displacement of merely 1 to 2 mm causes regional blood flow to precipitate to 18%–30% of baseline [10]. We hypothesize that the 6.0 mm threshold identified in our study may reflect a clinical proxy for a critical inflection point where neural tissue transitions toward sustained ischemic injury. However, as our study evaluated clinical outcomes and did not directly measure in vivo hemodynamics or histological damage, this mechanistic interpretation remains hypothesis-generating and requires further basic science validation.

In clinical practice, we have observed that the nerve root’s tolerance to retraction is substantially lower than its tolerance to compression. The physical metrics reflecting the extent of retraction primarily include time, distance, and force. Our study routinely recorded the cumulative retraction time and found that during standard PLIF procedures, retraction time was generally maintained at an average of approximately 10 min using an intermittent retraction technique. However, cumulative retraction time alone is limited as it cannot adequately differentiate the varying physiological impacts of sustained versus intermittent traction. We postulate that within this standard, well-controlled timeframe, the physical displacement—and the consequent neural tension—emerges as the decisive factor precipitating postoperative neurological symptoms.

Furthermore, the dose-response relationship identified in this study is markedly non-linear, with the risk escalating sharply when the retraction distance exceeds 8.0 mm. This pattern illustrates a distinct threshold effect in the neural tissue’s response to injury. Animal nerve retraction experiments by Byun et al. [30] demonstrated that 0.7 N serves as the critical limit for functional reversibility, whereas retraction forces exceeding 0.7 N cause irreversible structural and functional damage. Although basic experiments quantify the extent of retraction using absolute force, in actual clinical settings, an exponential increase in force invariably corresponds to an increase in the physical displacement of the retracted nerve root. This translation from biomechanical force to clinical displacement emphasizes the imperative for surgeons to exercise extreme caution when operative maneuvers approach this high-risk spatial zone.

The incidence of postoperative radiculitis observed in this study was 22.5%, which falls within the previously reported range of 0.9% to 44% [1–3]. The overall multivariable predictive model demonstrated modest discriminative ability (C-index = 0.739) and accounted for 26.3% of the outcome variance (Nagelkerke R^2^ = 0.263). Strikingly, even as a single parameter, the maximum L5 retraction distance achieved an area under the ROC curve (AUC) of 0.678. Given that postoperative neurological dysfunction is typically driven by a complex, multifactorial biomechanical process, the independent diagnostic efficacy of this single displacement parameter suggests its utility as a readily observable clinical indicator, although it does not fully encapsulate the complex, multifactorial mechanism of neural injury. This mechanical injury also translates into substantial long-term clinical consequences; 6-month follow-up results suggested an exploratory negative trend in the radiculitis group regarding return-to-work rates, patient satisfaction, and the need for additional interventions and reoperations. Given the limited sample size, these specific secondary endpoints are inconsistently powered and require cautious interpretation. Considering that over 60% of postoperative neurological deficits in lumbar surgery are attributable to L5 nerve root retraction [31], intraoperative monitoring of retraction distance as a direct and quantifiable parameter holds definitive clinical value.

While intraoperative neuromonitoring (IONM) remains a critical modality for assessing neural functional integrity, our quantitative strategy offers a complementary spatial safeguard with a low-cost barrier and robust universal applicability. Current literature indicates that the sensitivity and specificity of IONM for detecting nerve injury during lumbar surgery fluctuate significantly depending on the specific modalities and settings utilized [17, 18, 21, 22]. Rather than serving as a substitution for IONM, the 6.0 mm cutoff evaluated in our study provides immediate, proactive physical feedback. It demonstrates moderate overall diagnostic efficacy (sensitivity 66.7%, specificity 66.1%). While it yields a high negative predictive value (NPV) of 87.2%, the positive predictive value (PPV) remains limited at 36.4%. Clinically, this indicates that the 6.0 mm threshold is more effective for excluding low-risk cases (confirming relative safety when retraction is ≤ 6.0 mm) rather than reliably identifying which specific patients will develop radiculitis if the threshold is exceeded. It is important to note that the 6.0 mm cutoff was mathematically derived by maximizing the Youden index to balance diagnostic sensitivity and specificity. If surgeons prefer a more conservative safety margin to minimize missed risk, adopting a stricter alternative threshold of 5.0 mm would substantially increase sensitivity (from 66.7% to 88.9%) and NPV (to 91.3%), albeit at the cost of significantly reduced specificity (33.9%). Therefore, this threshold can be used alongside IONM to optimize intraoperative safety, or serve as an adjunctive reference tool to help stratify intraoperative risk in environments lacking IONM, rather than a definitive decision-making instrument. When operative maneuvers approach this 6.0 mm high-risk threshold, we strongly recommend proactive surgical adjustments, such as extending the lateral bony decompression to enlarge the operative corridor, employing intermittent retraction with regular relaxation phases, minimizing the duration of a single continuous retraction, or converting to a transforaminal lumbar interbody fusion (TLIF) approach. Ultimately, this intuitive, quantifiable strategy is particularly well-suited for widespread adoption in primary or grassroots healthcare facilities where expensive IONM equipment is largely unavailable.

While previous basic biomechanical studies have predominantly utilized absolute retraction force as the gold standard for evaluating microscopic neural injury, obtaining real-time, in vivo biomechanical data during clinical surgery typically necessitates expensive and complex customized sensing equipment. This practical barrier significantly restricts its widespread adoption, particularly in primary healthcare settings. To bridge this translational gap, our team’s preliminary in vitro biomechanical validation—utilizing advanced Fiber Bragg Grating (FBG) sensing technology—demonstrated a robust linear correlation between the transverse retraction distance of the nerve root and the mechanical tension it endures within a clinically relevant operational range (detailed data provided in Supplementary Materials). This biomechanical evidence substantiates that physical retraction distance can serve as a highly reliable surrogate metric for reflecting dynamic changes in microscopic neural stress. Translating this into a definitive 6.0 mm visual boundary within the surgical field provides surgeons of varying experience levels with a highly practical and easily adoptable safety monitoring protocol. Furthermore, establishing this quantifiable threshold lays a solid foundation for future large-scale clinical data collection and validation.

Several limitations of the present study should be acknowledged. First, constrained by the single-center retrospective design and a relatively limited sample size, the number of events per variable (EPV) in our multivariable logistic regression analysis was inherently low, posing a potential risk of model overfitting. We applied multiple validation steps to ensure these conclusions are robust. The DeLong test confirmed overall model efficacy, while maximizing the Youden Index pinpointed the optimal cutoff. Additionally, we used the Cochran-Armitage test to evaluate the dose-response trend and ran 1,000 bootstrap resamples to verify internal stability.

It is also important to recognize what the 6.0 mm mark actually represents: a statistical probability drawn from a specific cohort. Relying on a single displacement parameter naturally yields moderate diagnostic accuracy because patient anatomies and underlying pathologies vary wildly in practice. A patient with severe spondylolisthesis or extreme stenosis, for example, might experience critical nerve tension at just 4.0 mm of retraction. On the other hand, someone with highly compliant nerve roots could safely tolerate a much larger shift. Furthermore, retraction distance remains a partial surrogate; our measurements do not account for other critical variables that dictate neural injury, including the absolute applied force, the specific direction of traction, and individual baseline tissue susceptibility. Additionally, while we utilized a highly precise, pre-calibrated micrometer device to obtain objective physical readings and restricted the procedures to two senior surgeons to maintain technical consistency, we did not formally quantify inter- or intra-operator measurement reliability. Slight subjective variations in defining the zero baseline against the nerve root shoulder, particularly across varying anatomical complexities, could introduce unmeasured variability. Consequently, the required retraction distance functions not only as a direct physical exposure but also as an indirect marker of overall surgical complexity and anatomical narrowing (e.g., severe foraminal stenosis or epidural adhesions). Because we did not include specific radiographic or anatomical measurements as covariates in our multivariable model, residual confounding remains a distinct possibility; these underlying pathological factors may independently contribute to the development of postoperative radiculitis. Surgeons, therefore, should not treat 6.0 mm as an absolute rule. It functions better as a clinical red flag. Crossing this spatial boundary means entering a high-risk zone, requiring the surgical team to carefully weigh patient-specific intraoperative conditions and utilize neuromonitoring if available. Finally, the external generalizability of our findings is inherently limited. Because our cohort was strictly confined to single-level L4–5 PLIF procedures performed by two experienced surgeons, it remains uncertain whether the 6.0 mm threshold applies to other anatomical levels, multilevel fusions, revision cases, or alternative surgical approaches such as transforaminal lumbar interbody fusion (TLIF) and minimally invasive spine (MIS) techniques.

Moving forward, the integration of real-time biomechanical sensing into routine surgical retraction will be essential. Tracking the dynamic interplay between absolute retraction force and cumulative time will facilitate the construction of more precise, personalized predictive models tailored to varying spinal levels and complex pathological conditions.

## Conclusions

This study identifies a 6.0 mm statistically optimized risk threshold for transverse L5 nerve root retraction during posterior lumbar interbody fusion (PLIF). Exceeding this displacement limit significantly elevates the risk of postoperative radiculitis, translating into persistent pain, functional impairment, and an increased need for additional interventions at the 6-month follow-up. In conventional surgical environments lacking real-time in vivo biomechanical sensors, utilizing intuitive retraction distance as a reliable surrogate metric for neural stress offers a highly applicable and straightforward intraoperative safety strategy. By maintaining retraction at or below 6.0 mm, surgeons may potentially reduce the risk of iatrogenic nerve injury. Furthermore, this accessible physical monitoring approach lays a robust methodological foundation for future multicenter, large-cohort studies to validate its universal clinical applicability and provide rigorous evidence for precise nerve protection.

## Supplementary Information

Supplementary Material 1.

## Data Availability

The datasets generated and/or analysed during the current study are not publicly available due to institutional restrictions regarding patient privacy and clinical data protection, but are available from the corresponding author upon reasonable request.

## Abbreviations

PLIF: Posterior lumbar interbody fusion
DRG: Dorsal root ganglion
IONM: Intraoperative neuromonitoring
SSEPs: Somatosensory evoked potentials
s-EMG: Spontaneous electromyography
MEMS: Micro-electromechanical systems
FBG: Fiber Bragg Grating
MRC: Medical Research Council
VAS: Visual analog scale
ODI: Oswestry Disability Index
AIC: Akaike Information Criterion
VIF: Variance Inflation Factor
ROC: Receiver operating characteristic
AUC: Area under the curve
PPV: Positive predictive value
NPV: Negative predictive value
OR: Odds ratio
CI: Confidence interval
RR: Relative risk
NNH: Number needed to harm
PAR: Population attributable risk
TLIF: Transforaminal lumbar interbody fusion
EPV: Events per variable
