# Supplementary material for: Evaluating a 6.0-mm candidate warning level for intraoperative L5 nerve root retraction distance during posterior lumbar interbody fusion: a retrospective cohort study
Source: BMC Musculoskelet Disord. 2026 Aug 14;27:976. doi: 10.1186/s12891-026-10356-4 (PMC13757193; doi:10.1186/s12891-026-10356-4)
Supplement: Supplementary file 1 [file 12891_2026_10356_MOESM1_ESM.docx]

Supplementary Material

Supplementary Figure 1. In vitro biomechanical testing setup using a fresh-frozen sheep spine model.

Supplementary Figure 2. In vitro biomechanical calibration curve.

Supplementary Table 1. Multivariable logistic regression analysis using Firth’s penalized likelihood method.

**Supplementary Figure 1**. In vitro biomechanical testing setup using a fresh-frozen sheep spine model. The photograph demonstrates the custom micrometer-based retractor integrated with a Fiber Bragg Grating (FBG) sensor. This setup was utilized to simulate transverse nerve root retraction and accurately monitor the corresponding real-time mechanical tension.

**Supplementary Figure 2.** In vitro biomechanical calibration curve. The scatter plot illustrates the relationship between transverse L5 nerve root displacement (mm) and the corresponding wavelength shift (nm). Red solid circles represent the mean values at each 0.5-mm displacement increment, with black error bars denoting the standard deviation (SD) of three repeated trials. The solid red line indicates the linear regression fit, and the gray shaded area represents the 95% confidence interval. A strong linear correlation is observed (Pearson *r* = -0.90, *R*² = 0.814, *P* < 0.001).

Supplementary Table 1. Firth's penalized likelihood logistic regression model for sensitivity analysis

| Variables | Coefficient (β) | SE | OR (95% CI) | P value† |
| --- | --- | --- | --- | --- |
| Intercept | -10.387 | 3.626 | — | 0.002** |
| Preoperative VAS back pain | 0.600 | 0.382 | 1.82 (0.88–4.13) | 0.110 |
| Intraoperative blood loss | 0.016 | 0.011 | 1.02 (0.99–1.04) | 0.153 |
| **L5 root retraction distance** | **0.380** | **0.184** | **1.46 (1.02–2.16)** | **0.037*** |

**Notes:** β = Regression coefficient; SE = Standard Error; †*P* values were calculated using the profile penalized log-likelihood method (Likelihood ratio test = 10.038 on 3 *df*, *P* = 0.018). ‡Firth's penalized maximum likelihood estimation was applied to the AIC-optimized multivariable model to correct for potential small-sample and rare-event biases. After penalization, the overall model discrimination improved (C-index = 0.798; Brier score = 0.131)；Statistically significant difference (*P* < 0.05). ** Statistically significant difference (*P* < 0.01).
